# Use of COVID-19 Detection Tests in the Mexican Population from 2020-2022: Frequency and Distribution

**DOI:** 10.1590/0034-7167-2025-0301

**Published:** 2026-10-19

**Authors:** Irene Parada-Toro, Hortensia Reyes-Morales, Juan Espiniza-Montero, Sergio Flores- Hernández, Eric Monterrubio-Flores

**Affiliations:** I Instituto Nacional de Salud Pública Av. Universidad 655 Sta. María Ahuacatitlán. Cuernavaca, Morelos, México

**Keywords:** COVID-19, Serological Testing, Procedures and Techniques, Utilization, Public Health Services., COVID-19, Testes Serológicos, Procedimentos e Técnicas, Utilização, Serviços de Saúde Pública., COVID-19, Pruebas Serológicas, Procedimientos y Técnicas, Utilización, Servicios de Salud Pública.

## Abstract

**Objective:**

Analyze the frequency and distribution of COVID-19 diagnostic testing (RT-PCR and Ag-RDT) in Mexico during 2020-2022, and identify the sociodemographic and regional factors associated with their use.

**Methods:**

Ecological and cross-sectional study using secondary data from the National Epidemiological Surveillance System for Respiratory Diseases (SISVER). Approximately 15 million tests performed in public and private institutions between June 2020 and April 2022 were analyzed at individual and municipal levels to describe distribution by sex, age, social security coverage, and regional marginalization.

**Results:**

Ag-RDT was the most frequently used test nationwide, with testing concentrated in Mexico City and other economically developed regions, while marginalized and predominantly indigenous areas showed the lowest testing rates.

**Conclusion:**

Significant inequalities were found in access to COVID-19 testing in Mexico, highlighting the influence of socioeconomic and territorial disparities and the need for equity-oriented public health and nursing strategies.

## RESUMO

**Objetivo:**

Analisar a frequência e a distribuição dos testes diagnósticos de COVID-19 (RT-PCR e Ag-RDT) no México durante 2020 - 2022, e identificar os fatores sociodemográficos e regionais associados ao seu uso.

**Métodos:**

Estudo ecológico e transversal utilizando dados secundários do Sistema Nacional de Vigilância Epidemiológica de Doenças Respiratórias (SISVER). Aproximadamente 15 milhões de testes realizados em instituições públicas e privadas entre junho de 2020 e abril de 2022 foram analisados nos níveis individual e municipal para descrever a distribuição por sexo, idade, cobertura de seguridade social e marginalização regional.

**Resultados:**

O teste Ag-RDT foi o mais utilizado em nível nacional, com maior concentração de testagem na Cidade do México e em outras regiões economicamente desenvolvidas, enquanto áreas marginalizadas e predominantemente indígenas apresentaram as menores taxas de testagem.

**Conclusão:**

Foram identificadas desigualdades significativas no acesso aos testes de COVID-19 no México, evidenciando a influência das disparidades socioeconômicas e territoriais e a necessidade de estratégias de saúde pública e de enfermagem orientadas pela equidade.

## RESUMEN

**Objetivo:**

Analizar la frecuencia y la distribución de las pruebas diagnósticas de COVID-19 (RT-PCR y Ag-RDT) en México durante 2020-2022, e identificar los factores sociodemográficos y regionales asociados a su uso.

**Métodos:**

Estudio ecológico y transversal utilizando datos secundarios del Sistema Nacional de Vigilancia Epidemiológica de Enfermedades Respiratorias (SISVER). Se analizaron aproximadamente 15 millones de pruebas realizadas en instituciones públicas y privadas entre junio de 2020 y abril de 2022, a nivel individual y municipal, para describir la distribución por sexo, edad, cobertura de seguridad social y marginación regional.

**Resultados:**

La prueba Ag-RDT fue la más utilizada a nivel nacional, con una mayor concentración de pruebas en la Ciudad de México y en otras regiones económicamente desarrolladas, mientras que las zonas marginadas y predominantemente indígenas presentaron las tasas más bajas de realización de pruebas.

**Conclusión:**

Se identificaron desigualdades significativas en el acceso a las pruebas de COVID-19 en México, lo que evidencia la influencia de las disparidades socioeconómicas y territoriales y la necesidad de estrategias de salud pública y de enfermería orientadas a la equidad.

## INTRODUCTION

The COVID-19 global health crisis marked a turning point in public health, demanding an unprecedented focus on diagnostic testing as the cornerstone of disease control. Since October 2022, over 600 million cases of the virus have been recorded globally and six million deaths have been associated with SARS-CoV-2, demonstrating the pervasive impact of this disease^(1)^. However, the effects of the pandemic have been particularly severe in regions characterized by pronounced inequalities such as Latin America and the Caribbean (LAC), where pre-existing socioeconomic and public health disparities intensified the challenges posed by COVID-19. Even though it represents a small proportion of the world’s population, LAC accounted for approximately 13% of global infections and nearly 27% of deaths ^(1)^.

Endorsed by the World Health Organization (WHO) and the Pan American Health Organization (PAHO), large-scale diagnostic testing constituted an essential component of international strategies to mitigate transmission ^(2,3)^. Specifically, the Reverse Transcription Polymerase Chain Reaction Test (RT-PCR) and the Antigen-Detection Rapid Diagnostic Test (Ag-RDT) They established themselves as indispensable tools for identifying cases, interrupting chains of transmission, and guiding public health decision-making ^(3,4)^.

From the onset of the pandemic, COVID-19 testing proved particularly low in regions with a high proportion of populations living in vulnerable conditions. Various studies have shown that access to diagnostic and health services was unequal between and within countries, reflecting structural inequities rooted in the social and territorial determinants of health. In the United States, for example, these communities included Afro-Americans as well as individuals of low socioeconomic levels and lacking health-care coverage ^(5)^. In the LAC region, studies have found that population mobility did not drop during lockdowns, and diagnostic testing was delayed or limited in cities with highly marginalized areas ^(6)^. Furthermore, inequalities in service coverage and health system responsiveness have been documented to limit equitable access to diagnosis ^(7,8)^. These findings confirm that the region faces a widespread low testing rate, associated with fragmented systems and persistent socioeconomic gaps. In the case of Mexico, evidence points to a limited and heterogeneous diagnostic testing coverage, conditioned by socioeconomic status, institutional affiliation, and geographic location. Institutional reports have indicated that populations with lower levels of education, income, or access to health services experienced greater barriers to receiving timely care and diagnosis ^(9)^. Likewise, recent studies have shown that social and ethnic inequalities significantly influenced COVID-19 outcomes, and that mortality was higher in municipalities with greater social deprivation ^(10,11)^. Even official channels and international organizations early on recognized the insufficiency of diagnostic tests and the heightened risk for low-income populations and indigenous communities [UN News, 2020] ^(12)^.

To monitor and report COVID-19 cases, Mexico used the Sentinel Model, which had been adopted in conformity with international health strategies as part of the Mexican Epidemiological Surveillance System for Viral Respiratory Diseases (*SISVER*) ^(13)^. However, despite its compliance with international norms, the Organization for Economic Cooperation and Development (OECD) ranked Mexico among the countries with low testing rates owing to disparities in its testing frequency and distribution ^(14)^. The OECD study assessed the utilization of RT-PCR and Ag-RDT tests across Mexico based on socioeconomic factors and the number of health facilities, which reflected testing accessibility and uptake.

Despite the evidence, a knowledge gap persists regarding how COVID-19 tests were distributed according to sociodemographic and regional variables in Mexico. Therefore, this study aims to explore the utilization patterns of these tests.

## OBJECTIVE

Analyze the frequency and distribution of COVID-19 diagnostic testing (RT-PCR and Ag-RDT) in Mexico during 2020-2022, and identify the sociodemographic factors associated with their use.

## METHODS

An observational study, incorporating both ecological and individual-level cross-sectional analyses, was conducted following the STROBE guidelines. This secondary analysis utilized COVID-19 testing data (RT-PCR and Ag-RDT) from public and private health institutions, with analyses performed at the individual and municipal levels from June 2020 to April 2022. The study population comprised all individuals registered in the SISVER database in Mexico.

The analysis used datasets from the SISVER system, publicly available from the General Directorate of Epidemiology of the Mexican Ministry of Health (MOH), which contain individual-level records of COVID-19 diagnostic tests ^(15)^. The registry provides the result and date of the most recent test (RT-PCR or Ag-RDT) for each individual and aggregates data from outpatient, hospitalization, and death cases, as reported by 5,186 medical units. These units comprise 475 sites from the Sentinel surveillance system and 430 private facilities, encompassing all three levels of healthcare.

The SISVER system data were validated by CONAVE, the national oversight body with representation from federal, state, and municipal institutions. Records with missing values for the analyzed variables were excluded; these omissions constituted less than 0.001% of the total dataset.

Sociodemographic variables were analyzed at the individual and national levels, including: geographic region (categorized as 1=North, 2=Center, 3=Mexico City and its Metropolitan Area [Mexico City/MA], 4=South); state and municipality of residence (coded per the standard INEGI classification); age group (0-9, 10-19, 20-44, 45-64, 65-79, and ≥80 years); sex (1=Female, 2=Male); indigenous status, defined as indigenous-language speaker (0=No, 1=Yes); and degree of municipal marginalization (1=very high, 2=high, 3=medium, 4=low, 5=very low). At the institutional level, analyses assessed the sector of the health services performing the tests (public or private) and the Social Security coverage status of the populations served.

Testing rates were estimated per 1,000 people at the national level and stratified by region in Mexico: North, Center and South as well as Mexico City/MA. They were also disaggregated by municipality, sex, age group and degree of marginalization.

To analyze marginalization, we used the categories published by the National Population Council (*CONAPO*) ^(16)^. These, in turn, were taken from the Marginalization Index established by the National Statistics and Geography Institute (*INEGI)* according to the following indicators: being ≥15 years old and illiterate, lacking basic education, inhabiting a private household with no drainage, sanitation or electricity, or with dirt floors, living in an overcrowded dwelling, residing in a locality of <5,000 inhabitants, and being employed with an income of up to two minimum wages^(17)^.

Analysis took place in three phases. We first described testing frequency at the individual level by estimating proportions under the following categories: type of test administered (RT-PCR or Ag-RDT), age group, indigenous status (being an indigenous-language speaker), and type of health services applying the tests (private or public).

Second, we analyzed municipal-level data in order to estimate testing rate performed per 1,000 inhabitants. The rates were calculated for the entire Mexican population as well as by region, sex, age group, degree of marginalization and sector of the health services administering the tests (public or private). Data included the characteristics of the population groups tested.

During the third analytical phase, we constructed multiple linear regression models in order to analyze possible associations between the rates of testing rate performed at the municipal level and the sociodemographic and socioeconomic strata of the individuals tested. The latter served to identify “development poles,” that is, geographical areas marked by industrial and commercial activity generating economic expansion and social advancement ^(18)^. Estimations were based on the following indicators: total population, percentage of women, degree of municipal marginalization, number of health services about the Mexican Institute for Social Security (*IMSS*), region in Mexico, number of first-level health facilities and number of second-level health facilities.

After analyzing all the variables, we excluded those with either collinearity or no statistical significance. We performed quadratic transformations of the continuous variables in order to improve the associations and model fit. Means were adjusted using the marginal values of the models, with 95% CI. We ran the STATA V.15 statistical program in all three analytical phases ^(19)^.

Our work adhered to the principles of autonomy, confidentiality, beneficence and non-malfeasance. All analyses and assessments were based on information extracted from publicly accessible databases but following national and international ethical guidelines.

## RESULTS

Of the 15 million diagnostic tests administered in Mexico between 2020 and 2022, the RDP-A and Ag-RDT versions registered the largest and second-largest proportions of applications, respectively. The use of tests varied according to sociodemographic variables, with the highest rates recorded among individuals served by public health institutions, particularly those living in Mexico City/MA, aged 20 to 44 years and lacking health-care coverage. Table 1 presents additional sociodemographic characteristics of the study population.

**Table 1:** Demographic characteristics of Mexican population with used COVID-19 tests: México 2020-2021

Variable	Total		Women		Men	
	n	%	n	%	n	%
Both	788291	5.24	430414	5.36	357877	5.11
PDR-A	9416586	62.7	5094474	63.5	4322112	61.8
RT-PCR	4812882	32	2502898	31.2	2309984	33
North	3556432	23.7	1930212	24	1626220	23.3
Center	3046967	20.3	1652065	20.6	1394902	20
Mx City /AM	6010438	40	3197145	39.8	2813293	40.2
South	2403922	16	1248364	15.6	1155558	16.5
0 - 9	494625	3.29	231441	2.88	263184	3.76
10 - 19	1059205	7.05	545317	6.79	513888	7.35
20 - 44	8278310	55.1	4446636	55.4	3831674	54.8
45 - 64	4035362	26.9	2223355	27.7	1812007	25.9
65 - 79	919147	6.12	465522	5.79	453625	6.48
80 and more	231110	1.53	115515	1.43	115595	1.65
No	13899201	99.2	7432215	99.2	6466986	99.1
Yes	117411	0.83	61919	0.82	55492	0.85
Private	656049	4.36	329107	4.09	326942	4.67
With social security	5618125	37.4	301010	37.5	260801	37.3
Without social security	8743013	58.2	4688269	58.4	4054744	58.1

*Source: Own elaboration with data from the official website of the Federal Health Secretariat (SSA) of Mexico through the General Directorate of Epidemiology during the period 2020-2022.*

Table 2 presents the utilization levels and types of COVID-19 diagnostic tests, stratified by geographic region and institutional affiliation of the testing provider (public/private sector and Social Security coverage status). The Ag-RDT test was the most frequently used method, while RT-PCR was administered primarily in the South.

**Table 2:** Percentage of type of test used by region and health institutions tests used by region and health institutions

Type of test	PDR-A®		RT-PCR^©^®		Both types of tests		Total	
	n	%	n	%	n	%	n	%
^*^ **Country Region**								
North	1,956,583	55.0	1,403,121	39.5	196,728	5.5	3,556,432	100.0
Center	1,831,991	60.1	1,079,939	35.4	135,037	4.4	3,046,967	100.0
Mx City/MA	4,509,435	75.0	1,160,288	19.3	340,715	5.7	6,010,438	100.0
South	1,118,577	46.5	1,169,534	48.7	115,811	4.8	2,403,922	100.0
Total	9,416,586	62.7	4,812,882	32.0	788,291	5.2	15,017,759	100.0
**Type of Health Institutions**								
Private	417,599	63.4	235,152	35.7	6,314	1.0	659,065	100.0
Social Security	3,850,697	68.5	1376717	24.5	390711	7.0	5,618,125	100.0
SSA and Others	5,149,837	58.9	3,201,856	36.6	391,320	4.5	8,743,013	100.0
Total	9,418,133	62.7	4,813,725	32.0	788,345	5.2	15,020,203	100.0
**Social Security Health Institutions**								
IMSS	3,742,085	72.2	1,071,576	20.7	370,012	7.1	5,183,673	100.0
ISSSTE	92,184	29.0	210,609	66.2	15,331	4.8	318,124	100.0
PEMEX	6,347	10.6	50,906	84.7	2,879	4.8	60,132	100.0
Others^*^	10,081	17.9	43,626	77.6	2,489	4.4	56,196	100.0
Total	3,840,616	69.1	1,333,091	24.0	388,222	7.0	5,561,929	100.0
SSA and others Health Institutions^**^								
SSA	5,057,185	59.5	3070588	36.1	372,410	4.4	8,500,183	100.0
Other	90,621	37.8	130,342	54.4	18,851	7.9	239,814	100.0
Total	5,147,806	58.9	3,200,930	36.6	391,261	4.5	8,739,997	100.0

*Source: Own elaboration with data from the official website of the Federal Health Secretariat (SSA) of Mexico through té General Directorate of Epidemiology during the period 2020-2022.*

Regions of the country -North, Center, Mex City/MA (Mexico City and Metropolitan Area), South. IMSS: Mexican Institute of Social Security, ISSSTE: Institute of Security and Social Services of State Workers, PEMEX Medical Services, SSA: Health Secretary. PDR-A= Rapid Antigen Detection Test; RT-PCR= Real-time Reverse Transcription Polymerase Chain Reaction.

* Included SEDENA and SEMAR.

** without private Health Institutions.

Among individuals without Social Security coverage, the MOH conducted the highest number of tests. In contrast, the IMSS performed the largest volume for the population with such coverage. Both institutions predominantly utilized the Ag-RDT test. At the national level, the Petroleum Company Medical Services (PEMEX) and the Institute for Social Security and Services for State Workers (ISSSTE) employed the RT-PCR test more frequently than other Social Security institutions.

Table 3 presents the COVID-19 testing rates. Regionally, the highest rates were observed in the North and in Mexico City/MA. Regarding institutional performance, the private sector recorded the highest testing rate, despite serving a smaller number of users. In contrast, the Ministry of Health (MOH) achieved the highest absolute number of tests, attending to a significantly larger population. Disaggregated by sex, testing rates were higher among women. By age group, the highest rates were observed in the 45 to 64-year cohort, while the lowest were in the 0 to 9-year cohort.

**Table 3:** Distribution of rate x 1000 inhabitants by age groups, degree of marginalization and health institutions

Variable	Population	Total	Rate x1000
**National**	14949624	126000000	118.6
**Sex**			
Women	7986873	64540634	123.7
Men	6962751	61473390	113.2
**Age groups**			
0 - 9	491326	20811744	23.6
oct-19	1049662	21750230	48.2
20 - 44	8242914	47359785	174
45 - 64	4019933	25496965	157.6
65 - 79	915677	8106999	112.9
80 and more	230112	2214915	103.8
**Regions**			
North	3556388	32899542	108
Center	2978919	36960012	80.5
Mex City/A.C.	6010410	19313708	311.1
South	2403907	36687686	65.5
**Degree of Marginalization**			
Very high	46,729	3,517,600	13.2
High	172,581	7,894,980	21.8
Medium	360,186	9,370,743	38.4
Low	978,591	15,648,790	62.5
Very low	13,391,537	89,581,911	149.4
**Public and Private Services**			
Private	656037	2615213	250.8
Public	14307942	184200000	77.68

*Source: Own elaboration with data from the official website of the Federal Health Secretariat (SSA) of Mexico through the General Directorate of Epidemiology during the period 2020-2022.*

Figure 1A illustrates the distribution of testing rates across age and marginalization categories. Although individuals aged 60 and older were more vulnerable to SARS-CoV-2 infection and severe outcomes, the highest testing rate was observed among the 20-24 year-old cohort in the “very low” marginalization category. Conversely, within this same age group, the lowest testing rate was found among those in the “very high” marginalization stratum. A similar pattern was observed for the 45-64 year-old age group.

**Figure 1:**
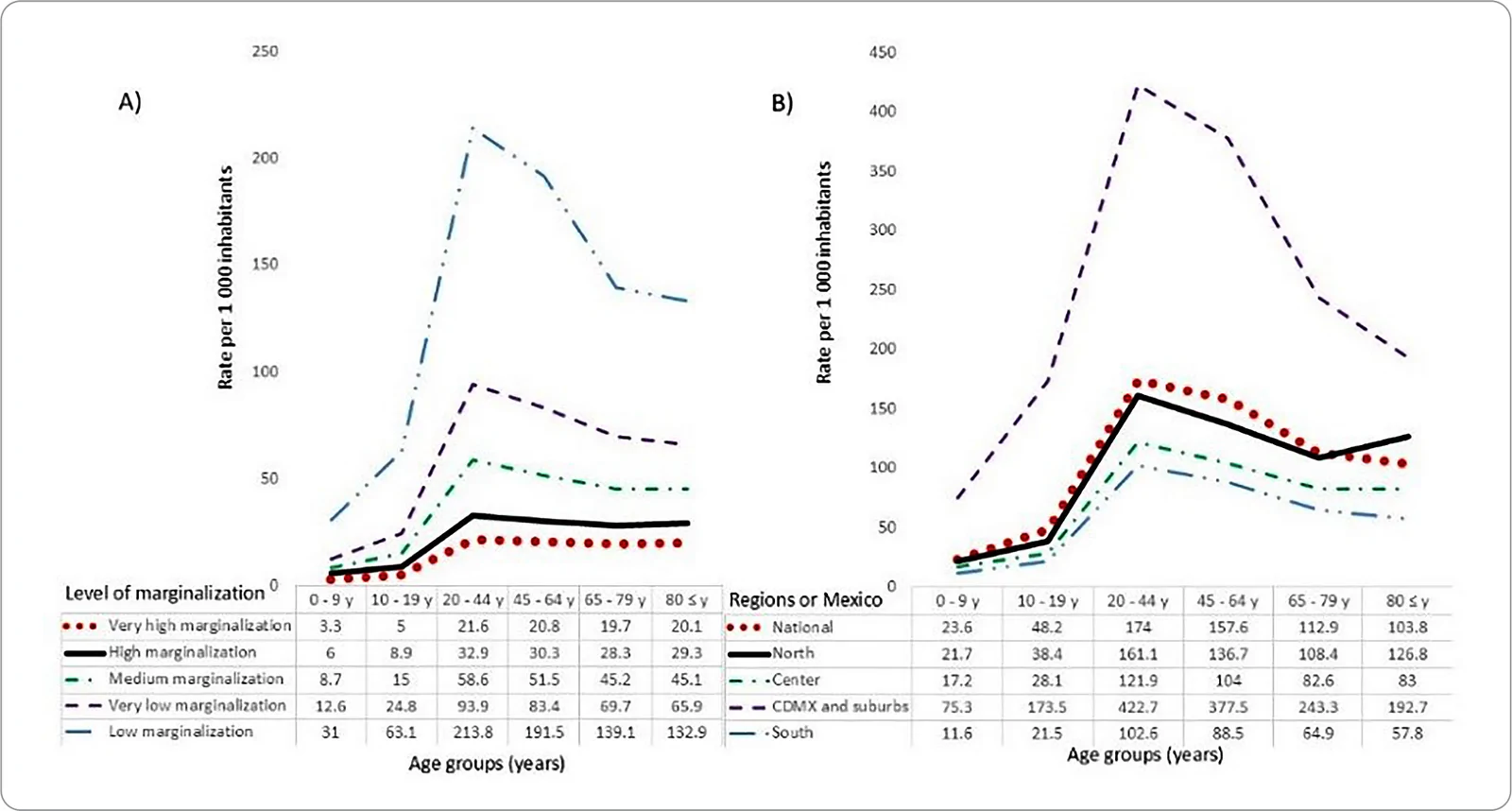
Rates by age group by level of marginalization and region of the country

*Source: Own elaboration with data from the official website of the Federal Health Secretariat (SSA) of Mexico through the General Directorate of Epidemiology during the period 2020-2022.*

*Model adjusted by Percentage of women, Percentage of women2, Marginalization index (ref. very high), High, Half, Low, Very low, Number of health services with social security, Number of health services with social security2, Regions of the country (ref. south), North, Center, CdMex /MA , Number of first level health services, Number of second level units.*

Figure 1B presents the distribution of testing rates adjusted by age and region. The highest age-standardized rates were observed among individuals aged 20-44, 45-64, and 80 years or older. Regionally, Mexico City/MA exhibited the highest rate, which was 2.4 times the national average, whereas the South reported a rate less than half of the national figure.

Table 4 presents the associations between testing rates and municipal-level indicators, including development pole markers. Bivariate analyses revealed significant positive associations for all variables (p < 0.001). The total population variable was excluded from the adjusted model due to collinearity with most other covariates. In the adjusted model, testing rates increased significantly with higher percentages of women (p<0.001) and a greater number of Social Security health services (p=0.049), and decreased with a rising marginalization index (p-trend <0.01). Compared to the South, rates were significantly higher in the North (p<0.001) and Mexico City/MA (p<0.001). Finally, a higher number of secondary-level health facilities was associated with increased testing (p<0.001).

**Table 4:** Lineal regression model of utilization rates at the municipal level with sociodemographic variables

Variables	Bivariate model				adjusted model		
	*β*	p	IC95%		*β*	p	IC95%
Total population	0.17	<0.001	(0.12, 0.22)		--	--	--
Percentage of women	0.74	<0.001	(0.56, 0.93)		1.05	<0.001	(0.78, 1.33)
Marginalization index (ref. very high)							
High	7.21	<0.001	(4.6, 9.83)		7.71	<0.001	(4.9, 10.53)
Half	17.75	<0.001	(14.67, 20.85)		15.24	<0.001	(11.74, 18.76)
Low	37.37	<0.001	(33.04, 41.72)		27.23	<0.001	(21.98, 32.5)
Very low	79.00	<0.001	(71.51, 86.51)		42.52	<0.001	(35.74, 49.32)
Number of health services with social security	10.46	<0.001	(8.25, 12.68)		2.64	0.0493	(0.01, 5.29)
Regions of the country (ref. south)							
North	42.46	<0.001	(36.66, 48.28)		16.67	<0.001	(10.98, 22.36)
Center	27.80	<0.001	(23.28, 32.32)		4.36	0.0728	(-0.4, 9.13)
MexCity /MA	287.00	<0.001	(209.31, 364.69)		202.00	<0.001	(128.99, 275.01)
Number units of first level health services	1.09	<0.001	(0.84, 1.36)		-0.03	0.8573	(-0.34, 0.28)
Number units of second level units	3.31	<0.001	(1.92, 4.71)		2.79	0.0004	(1.26, 4.32)
Percentage of women^2^	--	--	--		-0.01	<0.001	(-0.01, 0)
Number of health services with social security^2^	--	--	--		-0.02	<0.001	(-0.03, -0.01)
Constant	--	--	--		-25.20	<0.001	(-32.84, -17.56)

*Source: Own elaboration with data from the official website of the Federal Health Secretariat (SSA) of Mexico through the General Directorate of Epidemiology during the period 2020-2022.*

*Source: Own elaboration with data from the official website of the Federal Health Secretariat (SSA) of Mexico through the General Directorate of Epidemiology during the period 2020-2022.*

Figure 2 displays the adjusted probabilities of testing rates by sociodemographic characteristics and health service availability, based on the municipal-level linear regression model (Table 4). Panel A shows an inverse relationship between the level of marginalization and testing rates, where lower marginalization was associated with higher testing. Panel B shows that Mexico City/MA had the highest adjusted rate among all regions. Finally, Panels C and D demonstrate that municipalities with a greater number of primaryand secondary-level health care facilities exhibited the highest testing rates.

**Figure 2:**
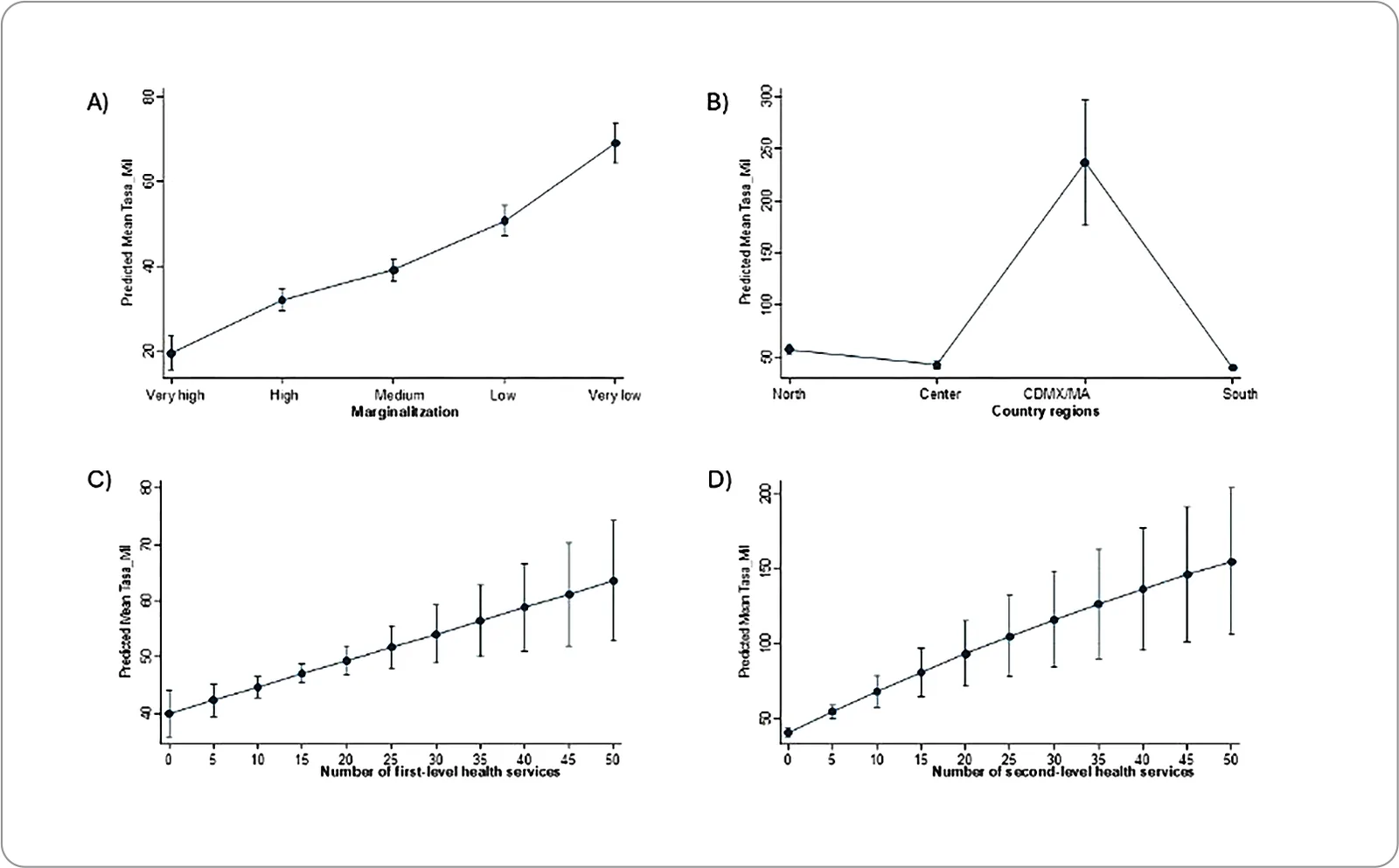
Use of adjusted probabilities estimated by a regression model at the municipal level

The following sociodemographic and institutional factors were associated with higher testing rates: residence in the North or Mexico City/MA, age 20 years or older, living in municipalities with very low marginalization levels, and having access to health services through the MOH or being affiliated with the IMSS.

## DISCUSSION

This study exposes the marked inequalities in the distribution of COVID-19 diagnostic tests (RT-PCR and Ag-RDT) in Mexico between 2020 and 2022. Testing was concentrated in regions with higher economic development and among the economically active population (20 to 64 years old), while areas with high marginalization and socially vulnerable groups recorded the lowest access rates.

These findings are consistent with the international literature. The low rates in municipalities with small populations and less infrastructure align with reports by Sepúlveda et al. (2023) ^(20)^, as well as with studies from European countries ^(21)^, the United States ^(22)^, and Latin America ^(7,8)^.

The relationship between economic development and COVID-19 testing described in our study is congruent with the findings of Mullachery ^(22)^, and other authors ^(23)^, who reported that the development of health infrastructure and the allocation of per-capita spending to institutions were influenced by the economic poles where they were located and the benefits they provided to their respective countries. Thus, a close relationship clearly exists between economic conditions and level of well-being, understanding the last as the complex interaction between health and quality of life.

Although countries with development levels similar to Mexico, such as Chile, Argentina, and Brazil, had significantly higher test utilization rates ^(24)^, their utilization patterns were equally determined by local economic development and infrastructure. Santos et al. (2023) note that this strategy widened inequality gaps, disproportionately affecting low-income populations and territories with less public investment, while Herrera et al. (2024) documented that interruptions in care were more frequent in households with lower educational attainment or without social protection.

The strong inverse association between municipal marginalization and testing rates constitutes one of the most relevant findings of the study. Municipalities with higher marginalization indices and less health infrastructure, particularly primary and secondary care services, had lower detection rates, consistent with the gradient of territorial vulnerability to COVID-19 described by Suárez et al. (2020) ^(25)^. In contrast, municipalities with low marginalization and greater installed health unit capacity recorded the highest testing rates, suggesting that the distribution of health resources, rather than epidemiological need, was the critical factor for diagnosis ^(23,26)^.

The higher utilization of tests was concentrated in the population aged 20 to 64, particularly among those with social security and formal employment. This pattern can be attributed to international recommendations that prioritized protecting productive sectors and the economically active population ^(27)^. Furthermore, our findings align with results published by Carnes Borrajo and Valenciano, as well as Trevisan et al., who also report that the majority of tests were conducted among the most active demographic in the labor market ^(28,29)^. Carnes Borrajo (2022) specifically indicates that Mexican labor policies during the pandemic prioritized the economic continuity of these productive groups. This economic orientation contrasts sharply with biological risk, as clinically vulnerable groups, such as the elderly and minors, were the least diagnosed phenomenon also observed in other countries where economic rationale prevailed over clinical risk criteria ^(30,31)^. Consequently, the lower diagnostic coverage among adults aged 65 and over underscores a structural disadvantage for populations outside the formal labor market and reinforces persistent inequalities in healthcare access.

The Ministry of Health (MOH) was the institution that performed the highest number of tests. This outcome can be explained by a federal policy, implemented in coordination with state authorities such as the Mexico City government, which was aligned with World Health Organization (WHO) recommendations from 2020. Under this policy, free testing services were provided at health-service facilities and health kiosks to all individuals, irrespective of their Social Security coverage, thereby expanding access to testing ^(32)^.

Concurrently, the IMSS-the institution with the most extensive population coverage and infrastructure-conducted the second-highest number of tests in Mexico ^(33)^. In 2020, consistent with the federal policy of providing free services during the health emergency, the IMSS expanded its services to the population without Social Security by establishing 3,843 Respiratory Care Modules (MARSS) ^(34)^.

At the national level, the Antigen Rapid Diagnostic Test (Ag-RDT) was the most commonly used method. An exception was observed in the Southeastern states, which recorded the highest percentage of RT-PCR testing. This regional disparity was influenced by the oil industry, a major driver of local economic development. The presence of a large proportion of the national oil company’s workforce, served by PEMEX Medical Services, in this region explains this distinctive pattern ^(34)^. Furthermore, several authors have reported that high contagion rates on oil platforms compelled PEMEX to revise its COVID-19 diagnostic strategy, replacing rapid tests with PCR tests^(35)^.

Analysis by region also revealed higher testing rates in Mexico City and its metropolitan area (MA). This can be attributed to its role as the country’s primary engine of economic development and a major contributor to the formal productive sector. The demographic profile of this region, characterized by a high concentration of adults aged 20 to 60 with Social Security coverage, further explains the observed testing intensity ^(36)^.

Nursing personnel established a foundational component within Mexico’s COVID-19 diagnostic framework during the pandemic. Their deployment proved instrumental in operationalizing both community health kiosks and the MARSS, where their scope of practice encompassed not only diagnostic test administration but also comprehensive patient triage, risk assessment, and case management, in accordance with federal health protocols (Secretaría de Salud, 2020). This multidisciplinary capacity at the containment frontlines-identified in epidemiological literature as a critical determinant of healthcare equity-was synergistically enhanced by their direct clinical care responsibilities. The nursing workforce thus emerged as the crucial nexus integrating diagnostic public health measures, clinical therapeutic interventions, and community-based healthcare delivery, demonstrating exceptional adaptability throughout the public health emergency ^(37,38)^.

### Limitations of the study

This study had several limitations that must be considered when assessing the results. Most important was the underreporting of diagnostic tests conducted as recorded in the database used for analysis, mainly those administered in private health services, as well as tests purchased in drugstores and other over-the-counter establishments. Another limitation concerned the fact that the data source did not include sufficient variables to allow for examining factors of interest at the individual level - in this context, primarily available health infrastructure - which could have been associated with the use of diagnostic tests. In addition, given the nature of the registry employed, the possibility of selection bias must be considered. Finally, we were unable to break down the analysis by type of employment (formal vs. informal). Informal employment is highly prevalent in urban areas and encompasses most of the working population.

### Contributions to the field of nursing, health or public policy

This study presents evidence on COVID-19 diagnostic test distribution by municipality. The results demonstrate that utilization was highest in the poles of development.

## CONCLUSION

The study reveals significant disparities in access to COVID-19 diagnostic testing in Mexico, characterized by a concentration of testing resources in economically developed regions and among the productive-age population, thereby exacerbating the vulnerability of marginalized groups. These findings underscore the imperative to strengthen diagnostic equity policies and enhance epidemiological surveillance systems. Furthermore, it is crucial to emphasize the essential role of nursing personnel, extended beyond hospital care to encompass the planning and implementation of control strategies, testing administration and follow-up, and community health education.

## Data Availability

Data available at: https://www.gob.mx/salud/documentos/datos-abiertos-152127
